# Terahertz Time-Domain Spectroscopy for Non-Contact Porosity Estimation and Hydration Assessment of Hardened Cement Paste

**DOI:** 10.3390/ma19132726

**Published:** 2026-06-25

**Authors:** Lidan Tian, Zhiguo Wang, Ya Chen, Wentao Zhang, Linhao Wang, Xiangyu Li

**Affiliations:** 1 College of Civil Engineering, Taiyuan University of Technology, Taiyuan 030024, China; 2 College of Environment and Safety Engineering, North University of China, Taiyuan 030051, China; 3 College of Architecture and Civil Engineering, Beijing University of Technology, Beijing 100124, China

**Keywords:** terahertz time-domain spectroscopy, cementitious materials, cement hydration, porosity estimation, non-contact testing, mercury intrusion porosimetry, effective medium theory, refractive index

## Abstract

This study presents a systematic terahertz time-domain spectroscopy (THz-TDS) investigation of hardened cement paste, framed as a complex-optical measurement in which the real and imaginary parts of the response probe distinct microstructural attributes. Transmission-mode measurements were made on pastes with water-to-cement (w/c) ratios of 0.3, 0.4, and 0.5 at curing ages of 7, 14, 28, and 56 days. The effective refractive index, obtained from the time-domain pulse delay (7, 28, and 56 days, paired with mercury intrusion porosimetry), correlates strongly and linearly with porosity over nine porosity-paired conditions spanning 15.1–30.4% (pooled R^2^ = 0.94, *p* < 0.001). In a quasi-static effective-medium framework—where the pores a re far smaller than the THz wavelength—this reflects the dependence of the effective permittivity on the solid volume fraction: the Bruggeman model outperforms the Maxwell–Garnett model, and all data fall within the Wiener bounds, lying close to the upper bound, indicating a continuously connected solid matrix with isolated pores. Cross-validated porosity estimation is reliable to within about ±2 percentage points (refractive-index uncertainty ±0.02–0.04). The absorption follows a power law (β ≈ 1.0–1.3) characteristic of disorder-activated vibrational absorption, in which the loss of long-range order in the amorphous C–S–H relaxes the crystalline selection rules and couples the THz field to the full vibrational density of states. The refractive index (structure-sensitive, governed by volume fraction) and the absorption (material-sensitive, governed by solid disorder; estimated loss tangent of order 0.1) thus form two complementary channels. Combining the THz-derived porosity with the Powers hydration model gives a degree of hydration consistent with literature ranges—an indirect comparison rather than direct validation. These results establish THz-TDS as a non-contact, non-ionizing technique for rapid porosity estimation and hydration assessment of cementitious materials.

## 1. Introduction

Portland cement is one of the most widely used binding materials in the construction industry [1]. The hydration of cement is a critical process during which anhydrous phases react with water to form products such as calcium silicate hydrate (C–S–H) and portlandite (Ca(OH)_2_) [2]. This process is accompanied by progressive reduction in porosity, which is a key parameter governing the strength, durability, and permeability of cement-based materials [2]. Understanding the quantitative relationship between microstructural evolution and macroscopic properties during hydration is therefore of considerable scientific and engineering significance.

The microstructure of hardened cement paste can be described as a two-phase composite consisting of a solid matrix (hydration products and unreacted cement) and a pore network filled with pore solution or air (after drying). The pore network includes gel pores (<10 nm, intrinsic to the C–S–H structure), capillary pores (10 nm to 10 μm, representing the remnants of water-filled space), and air voids (>10 μm, typically from entrapped or entrained air) [2]. The total porosity and pore size distribution evolve continuously during hydration as hydration products progressively fill the initially water-saturated capillary pore space. For ordinary Portland cement with w/c ratios of 0.3 to 0.5, the total porosity typically ranges from about 10% to 40% after 7 to 56 days of curing [2].

Several experimental techniques have been employed to characterize pore structure and monitor hydration. Conventional methods include electrical resistivity measurement [3], X-ray diffraction (XRD) [4], scanning electron microscopy (SEM) [5], mercury intrusion porosimetry (MIP) [6], and nitrogen adsorption [7]. While these methods provide valuable microstructural information, they are generally destructive, time-consuming, or incapable of real-time in situ monitoring. There is thus a growing demand for non-destructive and non-contact techniques that can rapidly assess the pore structure of cementitious materials.

Terahertz (THz) radiation, with frequencies between 0.1 and 10 THz (wavelengths 30 μm to 3 mm), occupies a spectral region between the microwave and infrared bands [8]. THz time-domain spectroscopy (THz-TDS) offers coherent detection, broadband coverage, and sensitivity to both amplitude and phase, enabling direct determination of the complex refractive index without resorting to Kramers–Kronig relations [9]. The technique has been successfully applied to various porous media, most notably pharmaceutical tablets, where the effective refractive index has been shown to correlate strongly with porosity [10,11,12,13,14,15]. Compared to near-infrared (NIR), mid-infrared (MIR), and Raman spectroscopy, THz radiation is less susceptible to scattering from sub-millimeter heterogeneities, making it well-suited for probing materials such as cement paste [9].

In particular, THz-TDS has been widely used for characterizing the porosity of pharmaceutical tablets. Bawuah et al. [11] demonstrated that the effective refractive index measured at THz frequencies correlates linearly with the porosity of microcrystalline cellulose compacts, with correlation coefficients exceeding 0.99. Markl et al. [13] extended this approach to functionalized calcium carbonate tablets and showed that THz pore structure analysis can be performed in milliseconds, making it suitable for in-line quality control. Shen [14] provided a comprehensive review of THz applications in pharmaceutical sciences, highlighting the advantages of THz radiation for non-destructive coating thickness measurement and porosity quantification. These studies establish a strong precedent for applying THz-TDS to other porous media, including cementitious materials.

The effectiveness of THz-TDS for porosity measurement derives from the sensitivity of the refractive index to the volume fractions of constituent phases. For a two-phase composite of solid and air, the effective refractive index is bounded by the Wiener limits and can be predicted by effective medium theories such as the Bruggeman or Maxwell–Garnett models [16]. Tuononen et al. [17] demonstrated the utility of the Wiener bounds for validating THz-derived permittivity values of pharmaceutical tablets, while Peiponen et al. [18] showed that THz pulse broadening can serve as an additional porosity indicator independent of sample thickness.

Despite these advantages, THz-TDS studies on cementitious materials remain limited. Ray et al. [19,20] employed THz spectroscopy to track hydration kinetics of tricalcium silicate and Portland cement. Dolado et al. [21] reported THz fingerprints of cement pastes at frequencies above 0.6 THz. In a related study, the present authors analyzed the dielectric constant–pore structure relationship of hardened cement paste using the General Effective Media (GEM) model [22]. The present work instead establishes the effective refractive index–porosity calibration, applies the Bruggeman and Maxwell–Garnett effective-medium models together with Wiener bounds for quantitative porosity estimation, analyzes the frequency-domain absorption spectra through a disorder-activated power-law model, and infers the degree of hydration via the Powers model. To date, this combination of systematic optical-parameter extraction, effective-medium-based porosity estimation, and hydration inference for hardened cement paste has not been reported.

Beyond these hardened-state studies, a growing body of work has used THz spectroscopy to probe the dynamic processes of cement setting and the behavior of water at the nanoscale. Goracci et al. tracked the setting dynamics of commercial cements over more than 24 h using non-destructive THz spectroscopy combined with neutron scattering, resolving structural evolution in the 1–30 nm range and linking it to changes in the collective low-energy vibrations of interfacial water [23]. Complementary studies of confined and interfacial water dynamics have shown that THz spectroscopy is sensitive to the transition from free water to confined interfacial water and chemically bound hydroxyl species during hydration [24]. These works establish that the THz response of cementitious systems is governed largely by the state and confinement of water, which provides the physical basis for the porosity sensitivity exploited in the present study of hardened paste.

The objectives of this study are (i) to accurately extract the THz optical parameters of hardened cement paste in both the time domain (effective refractive index) and the frequency domain (absorption spectra) with rigorous uncertainty quantification; (ii) to establish and assess quantitative relationships between the effective refractive index and porosity using linear regression, the Bruggeman and Maxwell–Garnett effective medium models, and Wiener bounds; (iii) to quantitatively assess the scattering contribution using Rayleigh theory; and (iv) to demonstrate the feasibility of estimating the degree of hydration from THz-derived porosity via the Powers model.

## 2. Experimental Methods

### 2.1. THz Time-Domain Spectrometer

**Figure 1:**
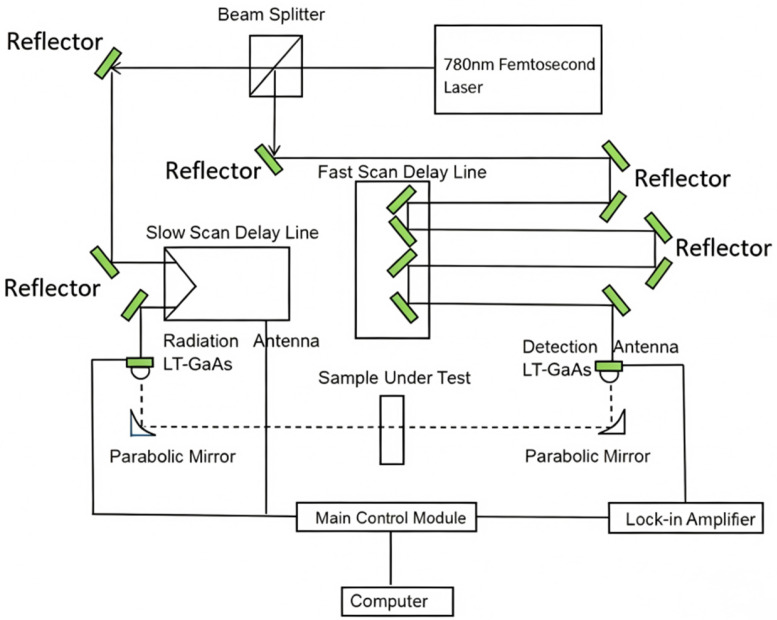
Schematic diagram of the transmission-mode THz-TDS system.

Measurements were performed using a transmission-mode THz-TDS system (Figure 1), which was custom-built by the team of Prof. Zhiyong Wang at Tianjin University (Tianjin, China) THz pulses were generated by a photoconductive antenna excited by a femtosecond laser and directed to pass normally through the central region of each sample. Three independent measurements were conducted per specimen, and the averaged time-domain waveform was used for analysis. The spectrometer was enclosed in a sealed chamber purged with dry nitrogen gas (relative humidity < 2.5%, temperature 23 ± 0.5 °C). The temporal step size was 0.02 ps.

The frequency resolution is inversely proportional to the total time window ΔT, which is determined by the travel range of the optical delay stage. The optical parameters of the sample were determined by comparing the THz pulse transmitted through the sample with a reference pulse recorded without a sample in the beam path. The THz beam diameter at the sample position was approximately 5 mm, ensuring that the beam passed through a homogeneous region of each disk sample.

### 2.2. Sample Preparation

**Figure 2:**
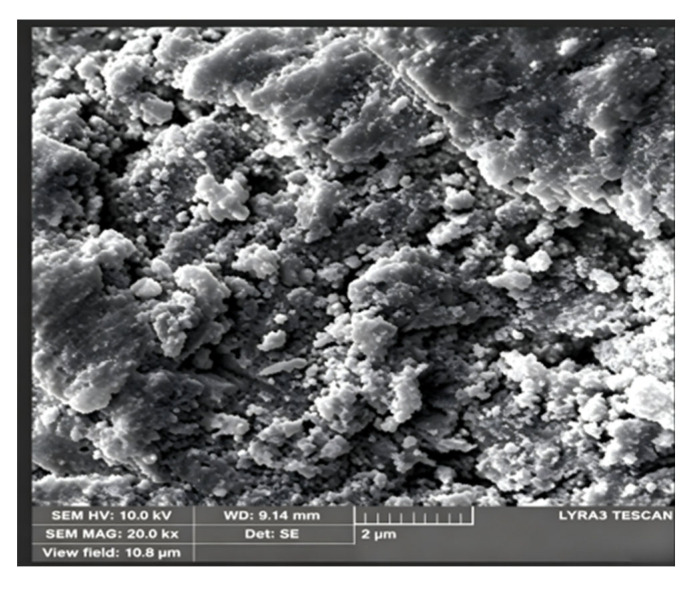
Representative SEM images of the polished sample surface.

Hardened cement paste samples were prepared with w/c ratios of 0.3, 0.4, and 0.5 using ordinary Portland cement (OPC, Type I) and cured under standard conditions for 7, 14, 28, and 56 days. The time-domain effective refractive index and the MIP porosity (7, 28, and 56 days) were reported previously [22] and are reanalyzed here with effective-medium models for porosity estimation; the frequency-domain absorption spectra (7, 14, 28 and 56 days) used for the power-law analysis (Section 4.3) are analyzed here for the first time. The cured blocks were sectioned into disks (~3 mm thickness) using a metallographic cutting machine with a diamond wheel, and both faces were ground and polished with 1200-mesh sandpaper at 750 r/min to minimize surface roughness [25]. The disk thickness was measured at five positions using a digital micrometer (precision ± 0.01 mm). After polishing, samples were immersed in anhydrous ethanol for 48 h to arrest hydration and then vacuum-dried at 60 °C to constant mass. SEM imaging (Figure 2) confirmed that residual surface defects were below 10 μm, far smaller than the THz wavelength (300 μm–3 mm at 0.1–1 THz).

The chemical composition of the ordinary Portland cement used in this study was determined by X-ray fluorescence (XRF) analysis. The major oxide components include CaO (63.5%), SiO_2_ (21.2%), Al_2_O_3_ (5.8%), Fe_2_O_3_ (3.1%), MgO (2.4%), and SO_3_ (2.8%). The Bogue phase composition yields approximately 56% C_3_S, 18% C_2_S, 9% C_3_A, and 9% C_4_AF. These values are typical of Type I ordinary Portland cement and are provided to facilitate comparison with other studies. The specific surface area (Blaine fineness) was 350 m^2^/kg.

Samples were prepared at three w/c ratios and four curing ages (7, 14, 28, and 56 days), with two to five specimens per condition (34 specimens in total) for repeatability assessment; the average thickness and standard deviation for each sample are reported in Table S1. All samples were stored in a desiccator over silica gel after vacuum drying and prior to THz measurement to prevent re-absorption of atmospheric moisture. The three w/c ratios (0.3, 0.4, 0.5) were selected to span the range most relevant to structural cementitious materials while producing a sufficiently wide porosity range (approximately 15–30%) for robust correlation; ratios below 0.3 are difficult to mix into homogeneous paste without admixtures, and ratios above 0.5 are uncommon in structural applications. The porosity-estimation analysis (Section 4.1, Section 4.2, Section 4.4 and Section 4.5) is based on the 7, 28, and 56-day samples, for which both MIP porosity and the time-domain effective refractive index are available (nine samples), while the absorption power-law analysis (Section 4.3) uses the frequency-domain spectra acquired at 7, 14, 28 and 56 days. Because two to five independent specimens were measured per condition, the specimen-to-specimen scatter of $n_{eff}$ (Section 4.1) provides a direct empirical estimate of the measurement reproducibility; a larger number of specimens would further tighten this estimate and is recommended for future work.

### 2.3. Mercury Intrusion Porosimetry

The porosity of each sample was determined by MIP (pore range: 3 nm to 1000 μm). Fragments were immersed in ethanol to stop hydration, vacuum-dried, and tested sequentially at low pressure (minimum 0.2 MPa) and high pressure (maximum 300 MPa). It should be noted that MIP has known limitations, including the ink-bottle effect and inability to detect closed pores [26]; these are discussed in Section 4.6.

The MIP instrument employed was an AutoPore IV 9500 (Micromeritics, Norcross, GA, USA). The contact angle of mercury was assumed to be 130°, and the surface tension was taken as 0.485 N/m. The Washburn equation was used to relate the applied pressure to the intruded pore diameter: d = −4γ cosθ/P, where d is the pore diameter, γ is the surface tension, θ is the contact angle, and P is the applied pressure. Representative cumulative and differential pore size distributions are shown in Table S2.

Table S2 also lists the total porosity measured by MIP for each sample. The porosity ranges from approximately 15.1% (w/c = 0.3, 56 days) to 30.4% (w/c = 0.5, 28 days), spanning a sufficiently wide range to establish meaningful correlations with THz optical parameters. The porosity generally decreases with hydration age at constant w/c ratio, as expected from the progressive filling of capillary pores by hydration products.

## 3. Data Processing Methodology

### 3.1. Time-Domain Parameter Extraction

**Figure 3:**
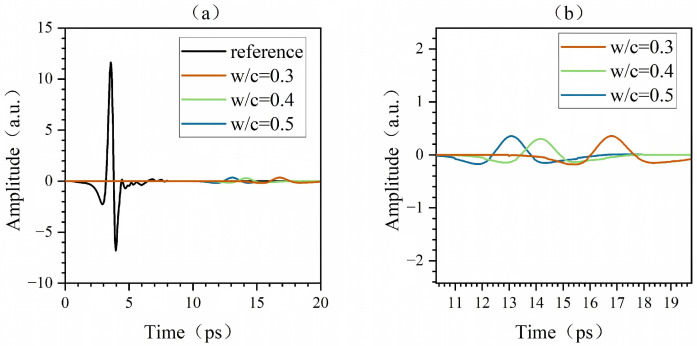
(**a**) Reference and sample THz time-domain waveforms. (**b**) Enlarged view of the sample signals showing amplitude attenuation and time delay.

Figure 3 presents the time-domain waveforms of cement paste samples (w/c = 0.3, 0.4, 0.5; 28 days) alongside the reference signal. The sample signals exhibit reduced amplitude and increased time delay relative to the reference. Amplitude attenuation increases with higher w/c ratios, while pulse delay increases with lower w/c ratios.

For a homogeneous slab with parallel faces, the effective refractive index is:

(1) $$n_{eff}=c\cdot \Delta t/d+n_{0}$$

where c is the speed of light in vacuum, d is the sample thickness, n_0_ ≈ 1.00027 is the refractive index of air, and Δt is the pulse delay relative to the reference.

This expression assumes that the sample is a homogeneous effective medium with negligible scattering, and that the pulse delay is measured between the peak positions of the reference and sample waveforms. The peak position was determined by fitting a parabola to the three highest points of each waveform to achieve sub-step temporal resolution. The resulting effective refractive index represents a volume-averaged value for the composite medium of solid matrix and air-filled pores.

For the present samples, the measured time delays range from approximately 10 ps (w/c = 0.5, 7 days) to 14 ps (w/c = 0.3, 56 days) for a sample thickness of ~3 mm. These delays correspond to effective refractive indices of 2.01 to 2.38. The variation in $n_{eff}$ across samples (approximately 18%) indicates appreciable sensitivity of the THz measurement to changes in porosity and solid-phase content. The measurement repeatability, assessed from the standard deviation of three independent measurements per sample, yields a coefficient of variation of approximately 1–2% for $n_{eff}$.

### 3.2. Frequency-Domain Parameter Extraction

The time-domain electric field was Fourier-transformed to obtain frequency-domain spectra. For a plane-parallel sample in dry atmosphere, the complex transmission coefficient including Fabry–Pérot reflections is [27]:

(2) $$T_{theory(\omega )}=P_{0}^{-1}T_{01}P_{1}T_{10}[1+{\sum (R_{10}^{2}P_{1}^{2})}^{i}]$$

The complex refractive index ñ = n + iκ was extracted by minimizing an error function based on both amplitude and phase of the transfer function [27]:

(3) Err=Σω[|ΔM(ω)|+|ΔA(ω)|]

where ΔM and ΔA are the amplitude and phase differences between theoretical and experimental transfer functions. Minimization was performed using the Nelder–Mead simplex algorithm [28] with convergence threshold 10^−4^.

Where the propagation terms are defined as $P_{0}=exp(i\omega n_{0}d/c)$ for the air reference path and $P_{1}=exp(i\omega ñH/c)$ for propagation through the sample of thickness d. The Fresnel transmission coefficients at normal incidence are $T_{01}=2n_{0}/(ñ+n_{0})$ and $T_{10}=2ñ/(ñ+n_{0})$, while the reflection coefficient is $R_{10}=(ñ-n_{0})/(ñ+n_{0})$. The summation over i accounts for multiple Fabry–Pérot reflections within the sample; for the present samples (thickness ~3 mm), the first echo arrives well outside the measurement time window, and i = 0 suffices.

In the absence of Fabry–Pérot reflections (i = 0), the theoretical transfer function simplifies to $T_{theory(\omega )}=[4ñn_{0}/{\left(ñ+n_{0}\right)}^{2}]\times exp[i\omega (ñ-n_{0})d/c]$. The magnitude of this expression yields the transmission amplitude, and its argument gives the phase shift. For optically thick samples where |T|<1/DR (below the noise floor), the extracted parameters become unreliable. This defines the upper frequency limit of the measurement, which was found to be approximately 0.7 THz for the 3 mm thick samples used here.

The error function formulation using both amplitude and phase (Equation (3)) ensures a unique solution for ñ at each frequency. This is a critical advantage over approaches that use only the real or imaginary part of the transfer function, which can yield periodic solutions due to the 2π ambiguity of the phase. Figure S2 in the Supplementary Material illustrates this point by showing the amplitude and phase error maps in the (n,κ) plane: while the amplitude map shows multiple local minima, the combined error function has a single global minimum.

The initial values for the optimization were estimated from the time-domain data as $\kappa_{1}=-(c_{0}/\omega d)ln(E_{s,max}/E_{r,max})$ and $n_{1}=c_{0}\Delta t/d+n_{0}$, where $E_{s,max}$ and $E_{r,max}$ are the maximal magnitudes of the sample and reference signals, respectively. These estimates provide a starting point sufficiently close to the global minimum to ensure convergence of the Nelder–Mead algorithm.

### 3.3. Signal Preprocessing

Signal preprocessing included: (i) time-domain averaging over three measurements [29,30]; (ii) zero-padding to improve frequency resolution; (iii) dynamic range analysis [31] to determine the effective bandwidth, yielding a reliable range of 0.3–0.7 THz; (iv) phase unwrapping initiated at 0.1 THz with linear extrapolation to lower frequencies; and (v) soft-threshold wavelet denoising using the Sym7 basis with five decomposition levels and Minimaxi threshold selection [32,33]. Details of these preprocessing steps are provided in the Supplementary Material.

The dynamic range (DR) was calculated as the ratio of the peak signal amplitude to the root-mean-square noise floor: $DR=E_{max}/N_{rms}$. The frequency-dependent DR determines the maximum measurable absorption coefficient via $\alpha_{max}\cdot d=2ln\{DR\cdot 4n_{s}/{\left(n_{s}+1\right)}^{2}\}$. Data beyond the frequency at which the measured absorption coefficient exceeds $\alpha_{max}$ were excluded from the analysis. Based on this criterion, the reliable frequency range for the present samples was determined to be 0.3–0.7 THz.

The optimal sample thickness for minimizing measurement uncertainty was estimated using the variance model of Withayachumnankul et al. [34]: $l_{opt}=2/\alpha (\omega )$. For the present samples, the estimated optimal thickness at 0.4 THz is approximately 1 mm. The actual thickness of ~3 mm was chosen as a compromise between spectral range and mechanical integrity required for accurate porosity determination by MIP.

Phase unwrapping was performed by adding integer multiples of 2π to the transfer function phase as needed. To avoid error propagation from the noisy low-frequency region, the unwrapping was initiated at 0.1 THz (where the signal-to-noise ratio is reliable) and linearly extrapolated to lower frequencies, with the phase constrained to start at 0 rad.

Wavelet denoising was applied using the Sym7 wavelet basis with five decomposition levels and the soft-threshold Minimaxi criterion. This procedure effectively suppresses oscillations caused by photon emission noise, thermal fluctuations, and residual scattering, while preserving the essential spectral features.

### 3.4. Measurement Uncertainty Analysis

The uncertainty in the effective refractive index arises primarily from the time-delay measurement (Δt) and the sample thickness (d). Using standard error propagation:

(4) $$\sigma (n_{eff})=n_{eff}\times \surd [{\left(\sigma_{\Delta t}/\Delta t\right)}^{\;2}+{\left(\sigma_{d}/d\right)}^{2}]$$

The time-delay uncertainty was estimated from the standard deviation of repeated time-delay determinations (typical $\sigma_{\Delta t}$ = 0.01–0.03 ps). The thickness uncertainty was determined from five-point micrometer measurements (typical $\sigma_{d}$ = 0.02–0.05 mm). The estimated uncertainty in $n_{eff}$ is ±0.02–0.04, corresponding to a relative uncertainty of approximately 1–2%.

### 3.5. Quantitative Assessment of Scattering

The Rayleigh scattering coefficient was estimated using [9]:

(5) $$\mu_{s}\approx N_{p}\cdot {\left(2\pi /\lambda \right)}^{4}\cdot a^{6}\cdot {\left|m^{2}-1\right|}^{2}/{\left|m^{2}+2\right|}^{2}$$

$\mu_{s}$ using representative parameters (critical pore diameter ≤ 80 nm from MIP, $n_{s}$ ≈ 2.6, φ ≈ 0.25), the scattering-to-absorption ratio/α is of the order of 10^−4^ to 10^−3^ at frequencies below 0.7 THz, confirming that scattering contributes less than 0.1% of the total attenuation. This validates the use of Beer–Lambert absorption and Fresnel transmission models for parameter extraction.

## 4. Results and Discussion

### 4.1. Effective Refractive Index and Porosity

Table 1 summarizes the effective refractive index values measured from the time-domain pulse delay for all samples. At each hydration age, $n_{eff}$ decreases monotonically with increasing w/c ratio, reflecting the higher porosity of samples with greater initial water content.

**Figure 4:**
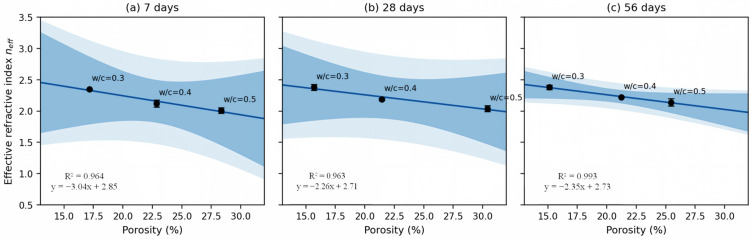
Effective refractive index vs. porosity of hydration, with linear fits, 95% confidence intervals (dark shading), and prediction intervals (light shading). Error bars represent ±1 standard deviation across the two to five replicate specimens per condition.

Figure 4 shows the effective refractive index as a function of MIP-measured porosity. A clear negative linear relationship between the effective refractive index and porosity is observed at every hydration age. Because each age contains only three w/c samples, the per-age fits are presented as illustrative trends rather than as independent significance tests; the consistently negative slope across all ages confirms the robustness of the trend. When all nine cement samples are pooled, the linear regression is highly significant, yielding R^2^ = 0.94 (*p* < 0.001), with slope = −2.53 ± 0.57 and intercept = 2.76 (95% confidence intervals). It should be noted that the porosity of the w/c = 0.5 sample at 28 days (30.4%) is slightly higher than at 7 days (28.4%), which reflects the inherent sample-to-sample variability of MIP measurements; this single point does not materially affect the pooled correlation. The corresponding per-age statistics and coefficient confidence intervals are listed in Table 2, and a residual analysis is provided in the Supplementary Material (Figure S7). The 95% confidence and prediction intervals are plotted as shaded bands in Figure 4. This linear dependence is consistent with findings for pharmaceutical tablets [11,13,15] and reflects the volumetric averaging of the refractive index over air-filled pores and the solid matrix.

Table 2 summarizes the linear regression statistics. The per-age fits use only three w/c samples each and are reported for completeness; with only one degree of freedom, the per-age fits do not reach statistical significance at the 5% level despite their high R^2^ values, reflecting the small number of points rather than a weak physical relationship. The pooled regression across all nine cement samples is highly significant (R^2^ = 0.94, *p* < 0.001) and provides the basis for the porosity calibration used in the remainder of this work. The pooled slope of −2.53 indicates that a 10 percentage-point increase in porosity reduces the effective refractive index by approximately 0.25. Given the measurement uncertainty of ±0.03 in $n_{eff}$, the minimum detectable porosity change is approximately 1–2 percentage points, which is sufficient for monitoring the hydration-induced porosity reduction in cement paste. Each of the nine porosity conditions was characterized by two to five independent specimens (34 specimens in total); the specimen-to-specimen standard deviation of $n_{eff}$ within a condition averaged 0.035 and never exceeded 0.06, below 10% of the 0.37 spread in $n_{eff}$ spanned by the porosity variation between conditions. The specimen-to-specimen scatter is thus small relative to the porosity-driven signal, confirming that the $n_{eff}$–porosity relationship reflects genuine microstructural differences rather than measurement noise and justifying the use of condition-averaged values in the regression.

In addition to amplitude attenuation and time delay, the THz pulse broadens with increasing porosity. This broadening is caused by scattering-induced diffusion of photons along irregular paths through the heterogeneous medium [18]. The full width at half maximum (FWHM) of the transmitted pulse increases with w/c ratio, suggesting that pulse broadening may serve as an independent, thickness-independent indicator of porosity. However, quantitative extraction of porosity from pulse broadening requires a more sophisticated scattering model and is left for future work.

The pore sizes in hardened cement paste (10 nm to 1 μm for capillary pores), the hydration product particle sizes (1 nm to 1 μm for C–S–H gel), and the unreacted cement particle sizes (1–50 μm) are all much smaller than the THz wavelength in the measurement range (300 μm to 3 mm at 0.1–1 THz) [1,35]. Consequently, the scattering is in the Rayleigh regime, as confirmed by the quantitative analysis in Section 3.5. Furthermore, the Kramers–Kronig analysis indicates low dispersion in this frequency range [36], consistent with the smooth, featureless absorption spectra reported in Section 4.3, which through the Kramers–Kronig relations imply a weakly dispersive refractive index.

### 4.2. Effective Medium Analysis

Effective medium theory provides a physical framework relating the optical properties of a composite medium to those of its constituents. For a two-phase system of solid cement matrix ($\varepsilon_{s}$) and air pores (ε_0_ ≈ 1), the Bruggeman and Maxwell–Garnett models are [16,37]:

(6) $$\mathrm{Bruggeman}:\;\;f_{S}(\varepsilon_{s}-\varepsilon_{eff})/(\varepsilon_{s}+2\varepsilon_{eff})+f_{0}(\varepsilon_{0}-\varepsilon_{eff})/(\varepsilon_{0}+2\varepsilon_{eff})=0$$

(7) $$\mathrm{MG}:\;\varepsilon_{eff}=\varepsilon_{s}[\varepsilon_{0}+2\varepsilon_{s}+2f_{0}(\varepsilon_{0}-\varepsilon_{s})]/[\varepsilon_{0}+2\varepsilon_{s}-f_{0}(\varepsilon_{0}-\varepsilon_{s})]$$

The Wiener bounds provide model-independent limits [16,17]:

(8) $$\varepsilon_{U}=f_{0}\varepsilon_{0}+f_{s}\varepsilon_{s};\varepsilon_{L}={\left(f_{0}/\varepsilon_{0}+f_{s}/\varepsilon_{s}\right)}^{-1}$$

**Figure 5:**
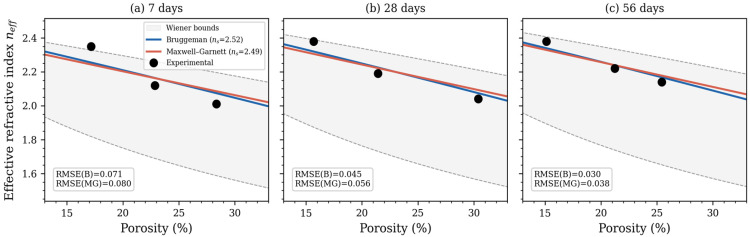
Comparison of experimental $n_{eff}$ vs. φ with predictions from the Bruggeman model, Maxwell–Garnett model, and Wiener bounds.

Figure 5 compares the Bruggeman model, Maxwell–Garnett model, Wiener bounds, and experimental data. The Bruggeman model provides a closer fit (lower RMSE) than the MG model, particularly at higher porosities where pore–pore interactions become significant. The relevant data is listed in Table 3. All experimental data fall within the Wiener bounds, confirming the physical consistency of the measurements. The data points lie closer to the upper (parallel) Wiener bound, consistent with a connected solid matrix containing isolated pores.

The extracted solid-phase refractive index ns from the Bruggeman model is approximately 2.5–2.6 across all hydration ages. This value represents a volume-weighted average of the intrinsic refractive indices of the constituent solid phases: unhydrated cement clinker, C–S–H gel, portlandite (Ca(OH)_2_), ettringite, and minor phases. The slight increase in ns with hydration age (from 2.52 at 7 days to 2.58 at 56 days) is consistent with the progressive densification of the solid phase as anhydrous phases convert to hydration products, although the change remains small, indicating that the effective THz refractive index of the solid skeleton is relatively insensitive to the degree of hydration.

The Wiener upper bound corresponds to a parallel arrangement of the constituent phases (electric field parallel to the layers), while the lower bound corresponds to a series arrangement (electric field perpendicular to the layers). In the context of cement paste, neither extreme geometry is physically realistic; the actual pore–solid arrangement is intermediate, which explains why the experimental data fall between the bounds. The proximity of the data to the upper bound suggests a microstructure in which the solid phase is continuously connected, with pores acting as isolated inclusions—consistent with the known microstructure of hardened cement paste, where the C–S–H gel forms a continuous matrix surrounding capillary pores [2].

It is instructive to compare the solid-phase refractive index $n_{s}$ extracted from the Bruggeman model with independently measured values. The refractive index of crystalline portlandite (Ca(OH)_2_) has been reported as approximately 2.3 at THz frequencies, while that of anhydrous tricalcium silicate (C_3_S) is approximately 2.9–3.0 [19,20]. The Bruggeman-extracted $n_{s}$ ≈ 2.5–2.6 falls between these values, consistent with a mixture of hydration products and residual anhydrous phases, providing independent support for the extraction procedure.

### 4.3. Frequency-Domain Absorption Spectra

**Figure 6:**
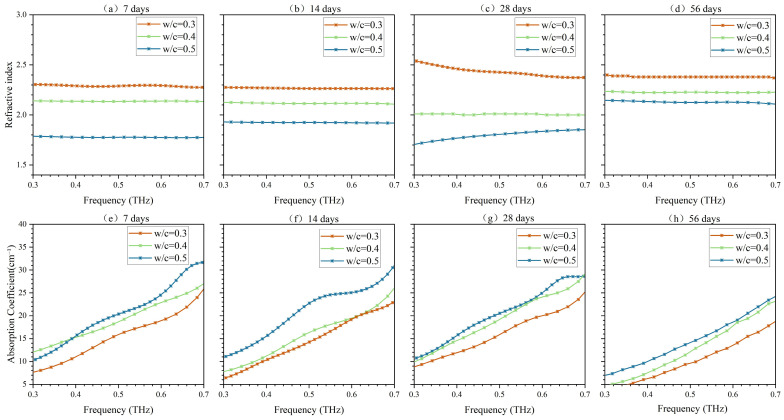
Refractive index and Frequency-domain absorption coefficient spectra of hardened cement paste of hydration in the 0.3–0.7 THz range.

Figure 6 shows the frequency-domain absorption coefficient spectra in the 0.3–0.7 THz range for samples cured 7, 14, 28 and 56 days. The absorption coefficient increases monotonically with frequency and with porosity (higher w/c ratio). No sharp spectral fingerprints were observed, consistent with the amorphous nature of C–S–H; the smooth, featureless absorption implies, through the Kramers–Kronig relations, a correspondingly weak dispersion of the refractive index over this band [36].

This weak dispersion underpins the use of the time-domain pulse delay to obtain the effective refractive index employed in the porosity analysis (Table 1, Section 4.1). When the extinction coefficient is small (κ ≪ n) and the unwrapped phase φ(ω) of the transfer function varies approximately linearly with frequency, the phase-derived refractive index n(ω) = φ(ω)c/(ωd) + 1 reduces to n(ω) ≈ cΔt/d + 1 = $n_{eff}$, where Δt is the group delay. The time-delay method (Equation (1)) therefore yields a representative, frequency-independent effective refractive index for these weakly dispersive samples, which is the quantity correlated with porosity in Section 4.1.

This contrasts with the findings of Dolado et al. [21], who reported absorption features at approximately 0.6 THz and 1.05 THz in cement pastes using density functional theory (DFT) simulations and experimental transmission measurements. The discrepancy can be attributed to several factors: (i) the present measurements are limited to below 0.7 THz, where any features near 0.6 THz may be too weak to resolve given the dynamic range of our system; (ii) the sample preparation differs significantly—Dolado et al. used sealed curing conditions, whereas our samples were ethanol-arrested and vacuum-dried, which may alter the C–S–H nanostructure and remove interlayer water responsible for some spectral features; and (iii) the polycrystalline nature of portlandite may contribute sharp phonon modes that are obscured in the heterogeneous paste. Extending the accessible bandwidth above 1 THz, potentially using pressed pellet samples with reduced thickness to maintain adequate dynamic range, could reveal additional spectral features in future studies.

The increase in absorption with porosity at a given frequency is not attributable to pore scattering, which is shown below to be negligible in this band; it more plausibly reflects intrinsic differences between pastes of different w/c ratio—principally bound water retained in the larger pore volumes of the higher-w/c pastes after drying—water being a strong THz absorber—superimposed on the disorder-activated vibrational absorption of the solid phase. Because the absorption spectra and the MIP porosity were acquired at different curing-age sets (7/14/28/56 days and 7/28/56 days, respectively), a pooled absorption–porosity regression across all samples is not warranted; for the two overlapping ages (7 and 28 days) the 0.4 THz absorption coefficient nonetheless decreases monotonically with decreasing porosity, indicating that absorption can serve as a qualitative, complementary porosity indicator. It is, however, less suitable than the refractive index for quantitative estimation: whereas the refractive index is governed by the pore volume fraction through the effective-medium response, the absorption reflects intrinsic, w/c-dependent properties of the solid phase that vary non-linearly with porosity, making it a less direct porosity probe. The fitted power-law exponents (β = 1.0–1.3) are far below the value of 4 expected for Rayleigh scattering. Because the pore sizes (0.01–10 μm) are two to four orders of magnitude smaller than the THz wavelength (0.43–1.0 mm), any pore scattering lies deep in the Rayleigh regime, where the scattering cross-section scales as (a/λ)^4^ ≈ 10^−12^–10^−8^ and is therefore negligible. The observed exponents instead match those expected for disorder-activated vibrational absorption in amorphous solids, indicating that the THz absorption is dominated by intrinsic vibrational loss in the solid hydration products rather than by pore scattering. This also explains why the scattering-insensitive refractive index is a more precise porosity indicator than the absorption coefficient.

It is informative to compare the absorption coefficients obtained here with those reported for pharmaceutical tablets at similar THz frequencies. Bawuah et al. [12] measured absorption coefficients of approximately 5–15 cm^−1^ at 0.4 THz for microcrystalline cellulose compacts with porosities of 10–30%, while Markl et al. [13] reported values of 8–20 cm^−1^ for functionalized calcium carbonate tablets [38]. The absorption coefficients of hardened cement paste (approximately 12–18 cm^−1^ at 0.4 THz) fall within a similar range, despite the very different chemical composition, suggesting that the dominant absorption mechanism in both cases is related to the disordered nature of the solid phase rather than to specific chemical bonds. Taken together, the real and imaginary parts of the THz response probe distinct microstructural attributes. The real part (the effective refractive index) is governed by the quasi-static effective-medium limit: because the pores are far smaller than the THz wavelength, the effective permittivity depends on the solid volume fraction but not on pore size or shape, so $n_{eff}$ reports the total porosity, which alone accounts for 94% of its variance (R^2^ = 0.94) with no additional pore-structure parameter required. We note that total porosity and critical pore diameter are themselves strongly correlated across the sample set (r ≈ 0.94), so the present data cannot statistically separate their individual contributions; the size-independence of $n_{eff}$ follows from the quasi-static regime (pore sizes ≪ THz wavelength) rather than from the regression. A practical corollary is that THz-TDS in this configuration quantifies total porosity (a volume-fraction measure) but cannot resolve pore size or pore-size distribution. The imaginary part (the absorption, equivalently the imaginary permittivity ε″ = 2nκ) is instead governed by the intrinsic vibrational disorder of the solid phase. Combining the two over the overlapping curing ages yields the complex dielectric function ε = ε′ + iε″. Using the measured absorption (Figure 6) at 0.4 THz as a representative estimate, κ = αc/(4πν) is of order 0.08–0.10, giving ε′ = n^2^ ≈ 4.0–5.7, ε″ = 2nκ of order 0.4, and a loss tangent tanδ ≈ 0.08–0.09; these values are indicative rather than precise, as the time-domain $n_{eff}$ and the frequency-domain absorption were acquired at partly different curing ages. The refractive index (structure-sensitive and scattering-insensitive) and the absorption (material-sensitive and disorder-driven) thus constitute two complementary channels: the former quantifies how much solid is present, the latter reflects the disordered, amorphous character of that solid. This dual sensitivity is what enables a single THz-TDS measurement to simultaneously estimate porosity and probe the nature of the hydration products.

The frequency dependence of the absorption coefficient was fitted to the disorder-activated absorption model [39,40]:

(9) $$n(\nu )\alpha (\nu )=K_{0}{\left(h\nu \right)}^{\;\beta }$$

As shown in Table 4, the exponent β ranges from 1.0 to 1.3, comparable to values reported for amorphous glasses (β ≈ 1.0–2.0) [39]. The coefficient K_0_ decreases systematically with increasing w/c ratio at each hydration age, reflecting the reduced solid-phase density.

The relatively low values of β (1.0–1.3) compared to crystalline materials (β > 2) reflect the high degree of structural disorder in the C–S–H gel, which is the dominant hydration product in hardened cement paste [2]. In the Strom–Taylor framework, β ≈ 1 corresponds to coupling of THz radiation to acoustic phonon modes through the relaxation of the crystalline selection rules by structural disorder [39]. The systematic decrease in K_0_ with increasing w/c ratio at each hydration age is consistent with the physical interpretation that K_0_ reflects the density of coupling modes per unit volume: higher porosity means less solid phase available for vibrational coupling, hence lower K_0_.

Notably, β does not show a clear systematic trend with either w/c ratio or hydration age, suggesting that the nature of the structural disorder in C–S–H gel does not change substantially over the hydration period studied (7–28 days). This is consistent with the observation that the C–S–H gel forms with a similar nanostructure regardless of the initial w/c ratio, while the total amount of gel (and hence the porosity) varies [2].

### 4.4. Porosity Estimation Accuracy

**Figure 7:**
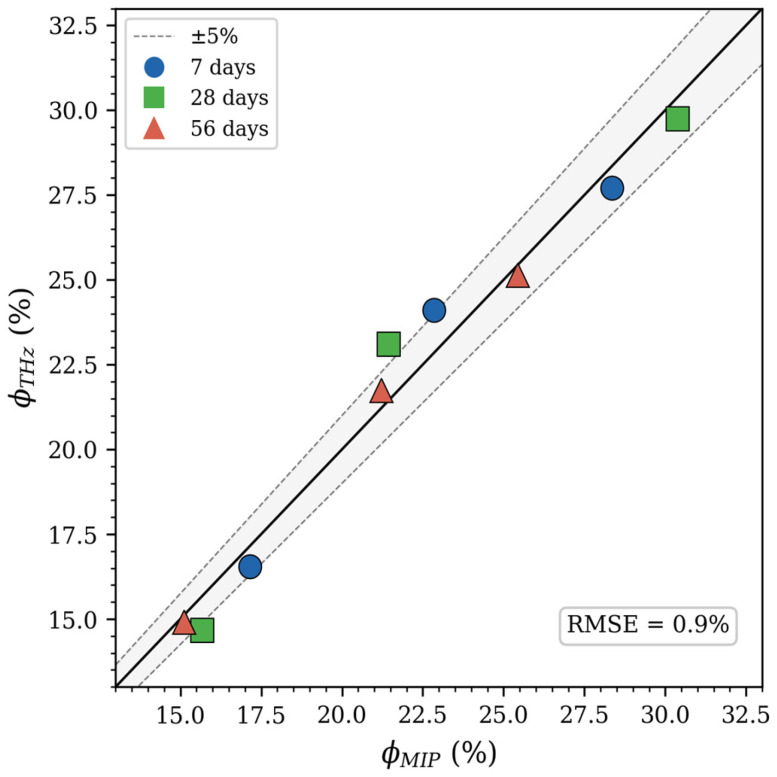
Parity plot: THz-predicted porosity vs. MIP-measured porosity. The solid line is 1:1; dashed lines indicate ±5% deviation.

To evaluate the practical utility of THz-TDS for porosity estimation, a leave-one-out cross-validation was performed: each sample was sequentially excluded, the linear calibration was refitted using the remaining samples, and the excluded sample’s porosity was predicted, listed in Table 5. Figure 7 shows the parity plot comparing THz-predicted and MIP-measured porosity.

The cross-validated results show that the porosity prediction error is distributed relatively uniformly across the porosity range, with no systematic bias toward over- or under-prediction. This indicates that the linear calibration model is appropriate for the range of porosities studied (15–30%). For samples with porosities outside this range, the calibration should be re-established with additional samples. The prediction accuracy could potentially be improved by using a separate calibration curve for each hydration age, as the solid-phase refractive index may vary slightly between ages due to differences in the relative proportions of anhydrous cement and hydration products. Propagating the measured refractive-index uncertainty (±0.03) through the calibration (slope dn/dφ = −2.53) yields a porosity uncertainty of approximately ±1.2 percentage points from the measurement alone; including the regression uncertainty, the THz-estimated porosity is reliable to within about ±1–2 percentage points over the 15–30% range studied, comparable to the typical reproducibility of MIP itself.

### 4.5. Hydration Degree Estimation via the Powers Model

A key advantage of non-contact porosity measurement is the ability to infer the degree of hydration (α) without destructive testing. The Powers model [2,41] provides:

(10) $$\varphi_{cap}=[(w/c)-0.36\alpha ]/[(w/c)+0.32]$$

By substituting the THz-estimated porosity, the degree of hydration can be estimated as:

(11) $$\alpha_{THz}=[(w/c)-\varphi_{THz}\cdot ((w/c)+0.32)]/0.36$$

**Figure 8:**
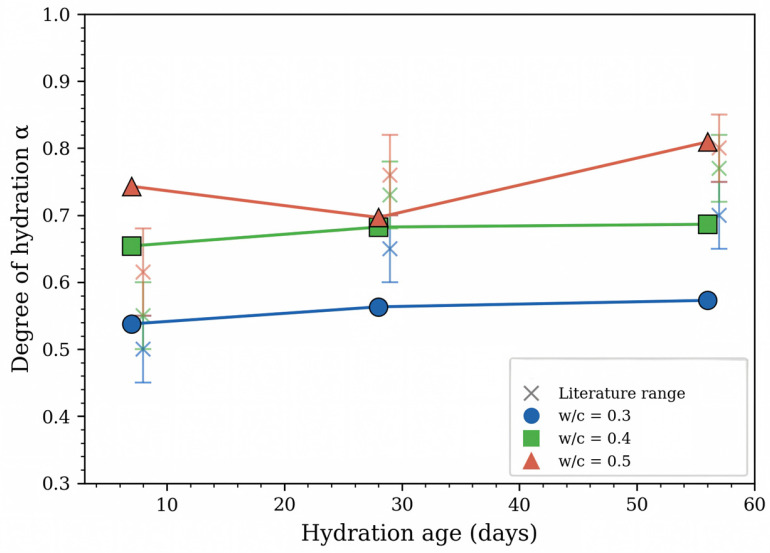
Degree of hydration estimated from THz-derived porosity via the Powers model (solid markers with connecting lines) compared with literature ranges (cross markers with error bars) for w/c = 0.3, 0.4, and 0.5.

Figure 8 compares the THz-estimated hydration degree with literature values for similar cement compositions, listed in Table 6. The αTHz values generally follow the expected trends—increasing with curing age and, at early ages, lower for low w/c ratios—and most fall within or close to the literature ranges. Some deviations are observed: the Powers model tends to overestimate α for high-w/c samples at early ages (e.g., w/c = 0.5 at 7 days) and underestimate it at later ages, reflecting the model’s assumption that all measured porosity is capillary porosity. These deviations support the feasibility of using THz-TDS as an indirect probe of hydration progress while underscoring that this is an indirect comparison rather than direct validation. The THz-derived hydration degrees are also in qualitative agreement with the nanoscale hydration dynamics reported in recent THz studies of setting cements [23,24], in which THz spectroscopy tracks the transition from free water to confined interfacial water and chemically bound hydroxyl species during cement setting.

### 4.6. Limitations and Practical Considerations

Several limitations of the present study should be acknowledged. The sample preparation procedure (cutting, polishing, ethanol immersion, and vacuum drying) is destructive and laboratory-based; reflection-mode THz measurements on as-prepared surfaces would be more practical for in situ applications and should be explored in future work. Moreover, because the specimens were dried, the measurements do not capture the moist, chemically evolving state of cement paste under real service or curing conditions, in which interaction with the surrounding medium can further alter the phase assemblage and pore structure [42]; extending the approach to such conditions is an important direction for future work.

The MIP reference method itself has well-documented limitations: the ink-bottle effect overestimates the proportion of small pores, the maximum mercury pressure does not access the full gel pore range (<3 nm), and high-pressure intrusion may damage fragile pore structures [26]. Future studies should therefore cross-validate MIP with complementary methods such as nitrogen adsorption or helium pycnometry.

The two-phase (solid + air) simplification ignores the possible presence of residual moisture in gel pores, which would increase the effective refractive index. In addition, the absence of spectral fingerprints in the 0.3–0.7 THz range limits chemical specificity. A principal direction of future work is therefore to extend the measurement bandwidth above 1 THz, where features associated with specific hydration products may appear, and to assign these features to individual phases (e.g., portlandite, ettringite, and C–S–H) by establishing quantitative correlations with phase-sensitive techniques such as TGA, XRD-Rietveld, and FTIR [21]. The present study is also based on nine primary sample compositions, and a larger sample set including different cement types would strengthen the generalizability of the calibration relationships. Most importantly, given the small number of samples per age, the hydration degree is inferred indirectly via porosity and the Powers model rather than measured directly; independent verification by TGA, XRD-Rietveld, or isothermal calorimetry—the established techniques for quantifying the degree of hydration [43]—is required to establish quantitative accuracy and is a priority for future work. The present 0.3–0.7 THz window, bounded by the dynamic range of the system, lies on the low-frequency flank of the vibrational spectrum, where the absorption follows a featureless power law; extending the bandwidth above 1 THz would additionally allow the reduced vibrational response to be examined for a boson peak, the hallmark of the disordered C–S–H network, which is expected at higher frequencies than are accessible here.

The effective medium analysis treats the cement paste as a simple two-phase composite (solid + air); in reality, hardened cement paste contains multiple solid phases with different refractive indices (unhydrated clinker, C–S–H, CH, ettringite), and the pore space may retain adsorbed water films even after vacuum drying. Although the Bruggeman inversion yields a solid-phase refractive index that remains stable at ≈2.5–2.6 across all ages (Section 4.2)—indicating that the pore volume fraction dominates the variation in $n_{eff}$—the contribution of the evolving phase assemblage to the THz response cannot be fully separated from that of the pore structure without complementary quantitative phase analysis such as XRD-Rietveld and thermal analysis [44,45]. A multi-phase effective medium model incorporating the volume fractions obtained from hydration calculations could provide a more accurate description and is recommended for future work.

Finally, the current transmission-mode geometry requires thin disk samples (~3 mm), which limits the measurement to laboratory-prepared specimens. For in situ evaluation of actual concrete structures, a reflection-mode configuration would be necessary. In reflection mode, the THz pulse reflects from the sample surface and the optical parameters are extracted from the reflected waveform. While reflection-mode THz-TDS has been successfully demonstrated for other materials [9], its application to rough concrete surfaces presents additional challenges due to surface scattering and the need for accurate surface reference measurements. Preliminary studies suggest that surface polishing or the use of a reference mirror in contact with the surface can mitigate these issues [25].

The reproducibility of the measurement should also be assessed more rigorously. While two to five replicate specimens were measured per condition and the specimen-to-specimen standard deviation was used to estimate uncertainty, a comprehensive inter-laboratory comparison or a larger statistical study with replicate samples would strengthen confidence in the reported precision and accuracy.

## 5. Conclusions

This study systematically investigated the THz optical properties of hardened cement paste using transmission-mode THz-TDS. The main conclusions are as follows:

(1) The effective refractive index was obtained from the time-domain pulse delay (7, 28, and 56 days) and the absorption coefficient spectra from the frequency-domain analysis (7, 14, 28 and 56 days) over the 0.3–0.7 THz band, with a measurement uncertainty of ±0.02–0.04 in $n_{eff}$.

(2) A strong linear correlation (pooled R^2^ = 0.94, *p* < 0.001) was established between $n_{eff}$ and MIP-measured porosity, with a consistently negative slope across all hydration ages. The Bruggeman EMT model outperforms the Maxwell–Garnett model in describing this relationship, and all data fall within the Wiener bounds.

(3) Quantitative Rayleigh scattering analysis confirms that the scattering contribution is negligible ($\mu_{s}/\alpha$ < 10^−3^) below 0.7 THz.

(4) The absorption spectra follow a power-law frequency dependence (β ≈ 1.0–1.3) consistent with disorder-activated vibrational absorption in amorphous C–S–H gel. The real (refractive index) and imaginary (absorption) parts of the THz response act as complementary probes—the former quantifying porosity via the volume-fraction-dependent effective-medium response, the latter reflecting the intrinsic vibrational disorder of the solid hydration products.

(5) By combining THz-derived porosity with the Powers hydration model, the degree of hydration was estimated and found consistent with literature ranges. We stress that this is an indirect comparison; direct validation by TGA, XRD-Rietveld, or calorimetry remains necessary to establish quantitative accuracy. With this caveat, the results demonstrate the potential of THz-TDS as a complementary tool for hydration assessment.

These findings establish THz-TDS as a promising non-contact technique for rapid porosity estimation and hydration assessment of cementitious materials. Future work should address measurements for field applicability and extended bandwidth for chemical identification.

From a practical standpoint, the method demonstrated here could be adapted for quality control of precast concrete elements, where rapid non-contact porosity assessment is valuable. The development of portable THz-TDS systems and the extension to reflection-mode geometry would further enhance the applicability of this technique to field conditions.

## Figures and Tables

**Table 1:** Effective refractive index and MIP porosity of all samples. Uncertainties represent one standard deviation.

Sample	w/c	Age (d)	$n_{eff}$ ± σ	$\varphi_{MIP}$ (%)
S1	0.3	7	2.35 ± 0.03	17.16
S2	0.4	7	2.12 ± 0.03	22.85
S3	0.5	7	2.01 ± 0.03	28.35
S4	0.3	28	2.38 ± 0.03	15.68
S5	0.4	28	2.19 ± 0.03	21.44
S6	0.5	28	2.04 ± 0.03	30.39
S7	0.3	56	2.38 ± 0.03	15.12
S8	0.4	56	2.22 ± 0.03	21.23
S9	0.5	56	2.14 ± 0.03	25.44

**Table 2:** Linear regression parameters for $n_{eff}$ vs. φ at each hydration age.

Age (d)	Slope a	Intercept b	R^2^	*p*-Value	95% CI (Slope)
7	−3.04	2.854	0.964	0.122	*n* = 3
28	−2.26	2.711	0.963	0.122	*n* = 3
56	−2.35	2.730	0.993	0.054	*n* = 3
Pooled	−2.53	2.758	0.940	<0.001	±0.57

**Table 3:** Quantitative comparison of Bruggeman and Maxwell–Garnett models.

Age (d)	$n_{s}$ (Brugg.)	$n_{s}$ (MG)	RMSE (Brugg.)	RMSE (MG)
7	2.52	2.49	0.071	0.080
28	2.57	2.54	0.045	0.056
56	2.58	2.55	0.030	0.038

**Table 4:** Power-law fitting parameters for absorption spectra.

Sample	β	K_0_	R^2^
7d, w/c = 0.3	1.279	11.64	0.990
7d, w/c = 0.4	1.225	9.48	0.973
7d, w/c = 0.5	0.997	10.28	0.996
14d, w/c = 0.3	1.157	11.04	0.956
14d, w/c = 0.4	1.217	8.38	0.989
14d, w/c = 0.5	1.260	6.54	0.997
28d, w/c = 0.3	1.173	11.68	0.979
28d, w/c = 0.4	1.245	8.52	0.997
28d, w/c = 0.5	1.336	6.76	0.957
56d, w/c = 0.3	1.255	4.00	0.995
56d, w/c = 0.4	1.121	4.38	0.995
56d, w/c = 0.5	1.245	1.08	0.999

The exponent β characterizes the frequency dependence of the absorption and is independent of the absolute value of the refractive index; the effective refractive index of each sample is reported in Table 1. K_0_ is the corresponding fitted coefficient.

**Table 5:** Comparison of THz-predicted and MIP-measured porosity.

Sample	w/c	Age (d)	$\varphi_{MIP}$ (%)	$\varphi_{THz}$ (%)	Rel. Error (%)
S1	0.3	7	17.16	16.55	3.6
S2	0.4	7	22.85	24.10	5.5
S3	0.5	7	28.35	27.71	2.2
S4	0.3	28	15.68	14.67	6.4
S5	0.4	28	21.44	23.09	7.7
S6	0.5	28	30.39	29.74	2.1
S7	0.3	56	15.12	14.91	1.4
S8	0.4	56	21.23	21.73	2.4
S9	0.5	56	25.44	25.14	1.2

**Table 6:** Degree of hydration estimated from THz-derived porosity via the Powers model.

Sample	w/c	Age (d)	$\varphi_{THz}$ (%)	$\alpha_{THz}$	$\alpha_{lit}$ (Typical)
S1	0.3	7	17.16	0.54	0.45–0.55
S2	0.4	7	22.85	0.65	0.50–0.60
S3	0.5	7	28.35	0.74	0.55–0.68
S4	0.3	28	15.68	0.56	0.60–0.70
S5	0.4	28	21.44	0.68	0.68–0.78
S6	0.5	28	30.39	0.70	0.70–0.82
S7	0.3	56	15.12	0.57	0.65–0.75
S8	0.4	56	21.23	0.69	0.72–0.82
S9	0.5	56	25.44	0.81	0.75–0.85

## Data Availability

The original contributions presented in this study are included in the article/Supplementary Material. Further inquiries can be directed to the corresponding author.

## Supplementary Materials

The following supporting information can be downloaded at: https://www.mdpi.com/article/10.3390/ma19132726/s1, Figure S1: Frequency-domain THz spectra; Figure S2: Error function landscape; Figure S3: Dynamic range analysis; Figure S4: Maximum measurable absorption coefficient; Figure S5: Phase unwrapping; Figure S6: Wavelet denoising; Figure S7: Regression residual analysis; Table S1: Sample dimensions; Table S2: MIP pore structure parameters.
