# Supplementary material for: Terahertz Time-Domain Spectroscopy for Non-Contact Porosity Estimation and Hydration Assessment of Hardened Cement Paste
Source: Materials (Basel). 2026 Jun 25;19(13):2726. doi: 10.3390/ma19132726 (PMC13362806; doi:10.3390/ma19132726)
Supplement: Supplementary file 1 [file materials-19-02726-s001.zip › materials-4376449-supplementary.pdf]

Article

Terahertz Time-Domain Spectroscopy for Non-Contact Porosity Estimation and Hydration Assessment of Hardened Cement Paste

Lidan Tian [1], Zhiguo Wang [1], Ya Chen [2], Wentao Zhang [1], Linhao Wang [1] and Xiangyu Li [3,*]

[1] College of Civil Engineering, Taiyuan University of Technology, Taiyuan 030024, China
[2] College of Environment and Safety Engineering, North University of China, Taiyuan 030051, China
[3] College of Architecture and Civil Engineering, Beijing University of Technology, Beijing 100124, China
* Correspondence: xiangyu@bjut.edu.cn

Supplementary materials

This section provides additional details on the signal preprocessing steps described in Section 3, including the frequency-domain spectra, error-function landscape, dynamic-range analysis, phase unwrapping, and wavelet denoising, together with the sample dimensions (Table S1), the MIP pore structure parameters (Table S2), and the regression residual analysis (Fig. S7).

Fig. S1. Frequency-domain THz spectra

The time-domain electric fields were Fourier-transformed to obtain the frequency-domain amplitude spectra shown below. The reference signal (black) exhibits a broad spectral peak centered near 0.8–1.0 THz, which defines the usable bandwidth of the spectrometer. The sample signals (colored lines) are significantly attenuated relative to the reference across the entire frequency range, with greater attenuation for higher w/c ratios. The amplitude ratio between sample and reference at each frequency defines the magnitude of the transfer function used for optical parameter extraction.

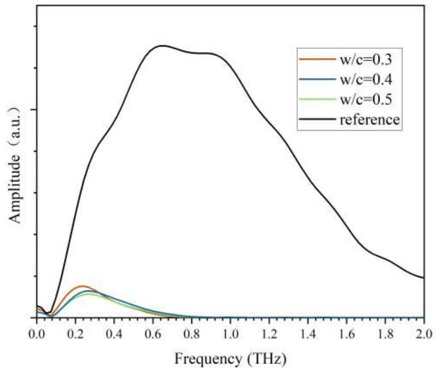

Figure S1. Frequency-domain amplitude spectra of the reference signal and sample signals (w/c = 0.3, 0.4, 0.5; 28-day samples). The reference spectrum peaks near 0.9 THz, while the sample spectra are strongly attenuated.

Fig. S2. Error function landscape

The optical parameters (n, κ) were extracted by minimizing the combined amplitude-phase error function (Eq. (3) in the main text). The figures below illustrate why the combined formulation is essential: using the real and imaginary parts of the transfer function separately yields multiple solutions (periodic roots), whereas the amplitude-plus-phase formulation produces a single global minimum.

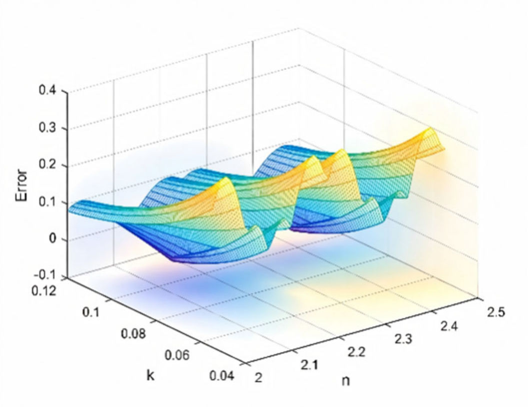

Figure S2. a). Three-dimensional error function landscape in the (n, κ) parameter space for the 28-day w/c = 0.4 sample at 0.35 THz, showing multiple local minima when using the real/imaginary formulation.

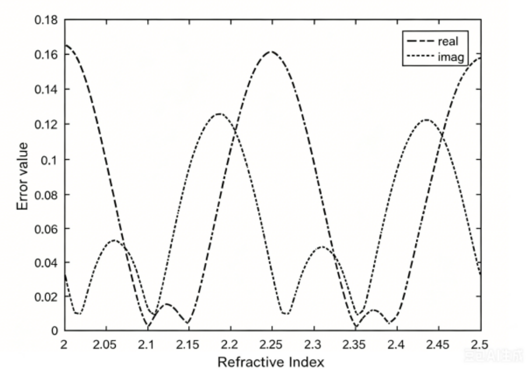

Figure S2. b). Error values as a function of refractive index n, showing periodic roots for the real （dased） par and imaginary （dot） part. The combined amplitude-phase error function avoids this ambiguity by yielding a unique solution at the intersection.

Fig. S3. Dynamic range analysis

The dynamic range (DR) of the THz-TDS system was determined from the ratio of the mean signal amplitude to the noise floor (dashed line). The DR decreases with increasing frequency due to reduced source intensity and increasing sample absorption. Reliable optical parameter extraction requires the signal amplitude to remain well above the noise floor, which limits the effective bandwidth to approximately 0.3–0.7 THz for the 3 mm thick cement paste samples used in this study.

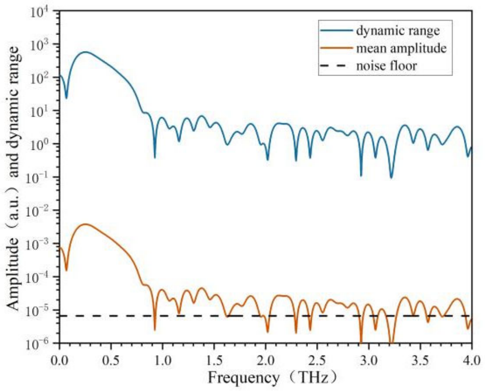

Figure S3. Frequency-dependent amplitude (red), noise floor (dashed), and dynamic range (blue) of the THz-TDS system.

Fig. S4. Maximum measurable absorption coefficient

The maximum absorption coefficient α_max that can be reliably extracted is determined by the dynamic range via Eq. (5) in the main text. Below the crossover frequency (where the measured α approaches α_{max}), the extracted optical parameters are reliable. Above this frequency (marked as the bandwidth limit), the measurement becomes noise-dominated. For the present samples, this analysis yields an effective bandwidth of 0.3–0.7 THz.

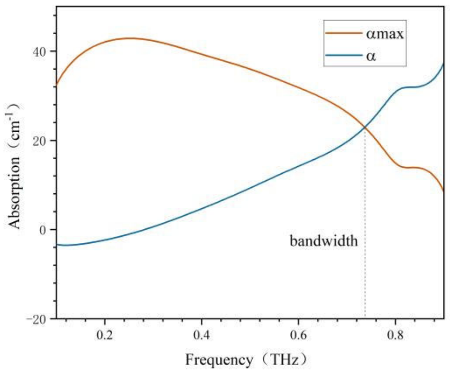

Figure S4. Maximum measurable absorption coefficient α_max (red curve) compared with the measured absorption spectrum α (blue curve) for the 28-day w/c = 0.4 sample. The vertical dashed line indicates the upper frequency limit of reliable measurement (~0.7 THz).

Fig. S5. Phase unwrapping

The phase of the transfer function, obtained from the ratio of sample to reference Fourier transforms, contains 2π discontinuities that must be unwrapped before optical parameter extraction. Fig. S5(a) shows the raw (wrapped) phase spectra, which oscillate between $-\pi$ and $+\pi$. After unwrapping, the phase decreases monotonically with frequency (Fig. S5(b)). The low-frequency region (below 0.1 THz) has insufficient signal-to-noise ratio for reliable phase measurement; therefore, the phase was linearly extrapolated from the 0.1 THz value back to zero frequency, with the constraint that $\varphi(0) = 0$.

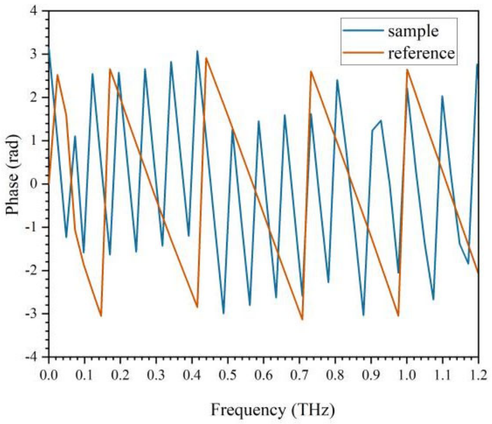

Figure S5. a). Raw phase spectra of the reference (red) and sample (blue) signals, showing 2π wrapping.

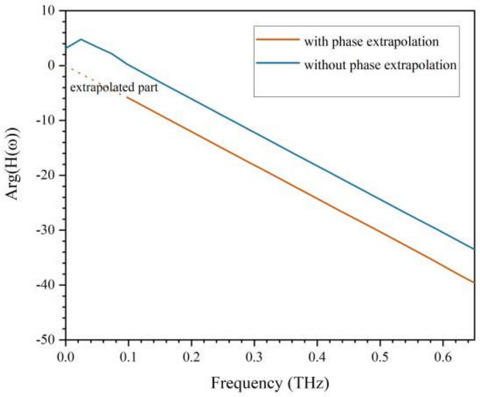

Figure S5. b). Unwrapped phase of the transfer function with (red) and without (blue) phase extrapolation below 0.1 THz. The dotted red line indicates the linearly extrapolated region.

Fig. S6. Wavelet denoising

Noise in the extracted refractive index and absorption coefficient arises from photon emission fluctuations, thermal noise, and residual scattering effects. A soft-threshold wavelet denoising procedure was applied using the Sym7 wavelet basis with five decomposition levels and the Minimaxi threshold selection criterion [29,30]. Fig. S6 shows the refractive index and absorption coefficient spectra before (blue) and after (red) denoising for the 28-day w/c = 0.4 sample. The denoising effectively suppresses high-frequency oscillations while preserving the spectral trends. The denoised data were used for all quantitative analyses in the main text.

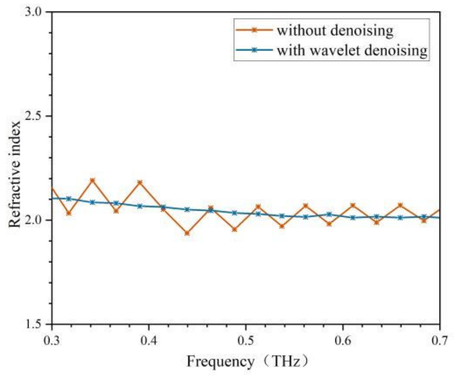

Figure S6. a). Refractive index spectra with (blue) and without (red) Sym7 wavelet denoising for the 28-day w/c = 0.3 sample.

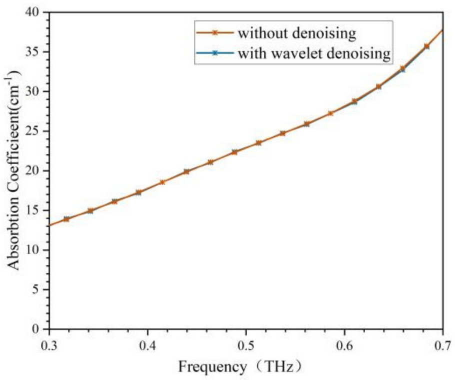

Figure S6. b). Absorption coefficient spectra with (blue) and without (red) Sym7 wavelet denoising for the same sample.

Fig. S7. Regression residual analysis

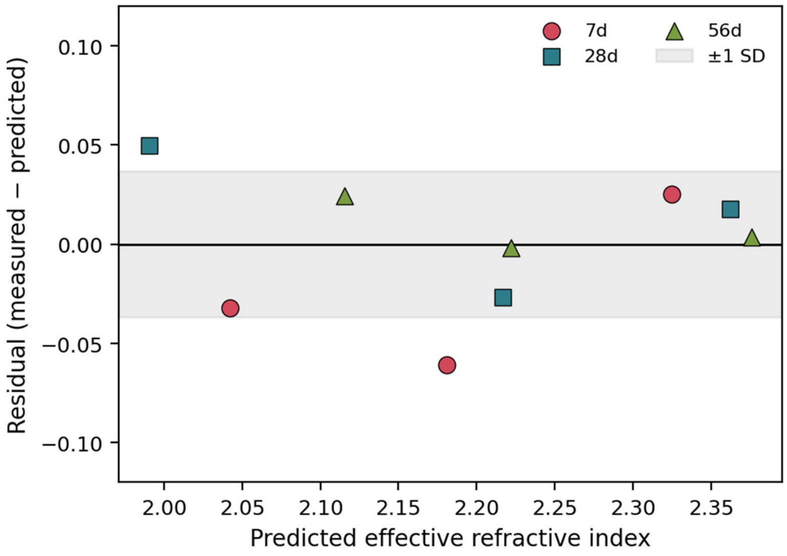

Figure S7. Residuals of the pooled effective refractive index–porosity regression (measured minus predicted n_{eff}) plotted against the predicted refractive index, by curing age. The residuals scatter

about zero with no systematic trend and lie within ±1 standard deviation (0.037), indicating that the linear calibration is unbiased across the porosity range studied.

Table S1. Sample dimensions

The thickness of each disc sample was measured at five positions using a digital micrometer (precision ±0.01 mm). The mean thickness and its standard deviation are listed below. These values were used for optical parameter extraction via Eqs. (1)–(3) in the main text.

Age（d）	w/c	Sample	Average thicness (mm)	Standard deviation(mm)
7d	0.3	1	3.03	0.012
		2	3.03	0.020
		3	2.99	0.018
	0.4	1	2.79	0.018
		2	3.05	0.025
		3	3.18	0.022
	0.5	1	2.70	0.023
		2	3.00	0.020
		3	2.89	0.010
14d	0.3	1	3.33	0.031
		2	3.35	0.023
		3	3.36	0.015
	0.4	1	2.86	0.025
		2	3.00	0036
		3	3.35	0.027
	0.5	1	2.79	0.023
		2	3.07	0.028
		3	3.15	0.015
28d	0.3	1	3.46	0.025
		2	3.52	0.026
		3	3.57	0.039
	0.4	1	2.91	0.020
		2	3.03	0.025
		3	3.25	0.024
	0.5	1	3.23	0.020
		2	3.32	0.011
		3	3.61	0.012
56d	0.3	1	3.18	0.012
		2	3.24	0.022
		3	3.25	0.018
	0.4	1	3.32	0.018
		2	3.56	0.025
		3	2.96	0.032
	0.5	1	3.56	0.023
		2	3.34	0.025
		3	3.12	0.011

Table S2. MIP pore structure parameters

Mercury intrusion porosimetry was performed using an AutoPore IV 9500 (Micromeritics, USA). The total porosity, critical pore diameter, total pore surface area, and

total pore volume for each sample are listed below. Representative cumulative and differential pore size distribution curves can be provided upon request.

Table S2. The total porosity, critical pore diameter, total pore surface area, and total pore volume for each sample by MIP test.

Age (d)	w/c	Porosity (%)	Critical pore diameter (nm)	Pore surface area （m^2/g）	Pore volume (ml/g)
7d	0.3	17.16	26.23	11.69	0.087
	0.4	22.85	50.25	17.28	0.13
	0.5	28.35	77.05	20.15	0.17
28d	0.3	15.68	26.24	15.38	0.086
	0.4	21.44	62.41	25.30	0.13
	0.5	30.39	76.97	32.16	0.21
56d	0.3	15.12	32.32	12.79	0.081
	0.4	21.23	50.26	23.84	0.13
	0.5	25.44	76.95	29.53	0.17
